# The Mood-Stabilizer Lithium Prevents Hippocampal Apoptosis and Improves Spatial Memory in Experimental Meningitis

**DOI:** 10.1371/journal.pone.0113607

**Published:** 2014-11-19

**Authors:** Fabian D. Liechti, Nicolas Stüdle, Regula Theurillat, Denis Grandgirard, Wolfgang Thormann, Stephen L. Leib

**Affiliations:** 1 Neuroinfection Laboratory, Institute for Infectious Diseases, University of Bern, Bern, Switzerland; 2 Graduate School for Cellular and Biomedical Sciences, University of Bern, Bern, Switzerland; 3 Clinical Pharmacology Laboratory, Institute for Infectious Diseases, University of Bern, Bern, Switzerland; 4 Biology Division, Spiez Laboratory, Swiss Federal Office for Civil Protection, Spiez, Switzerland; Universidade do Extremo Sul Catarinense, Brazil

## Abstract

Pneumococcal meningitis is associated with high morbidity and mortality rates. Brain damage caused by this disease is characterized by apoptosis in the hippocampal dentate gyrus, a morphological correlate of learning deficits in experimental paradigms. The mood stabilizer lithium has previously been found to attenuate brain damage in ischemic and inflammatory diseases of the brain. An infant rat model of pneumococcal meningitis was used to investigate the neuroprotective and neuroregenerative potential of lithium. To assess an effect on the acute disease, LiCl was administered starting five days prior to intracisternal infection with live *Streptococcus pneumoniae*. Clinical parameters were recorded, cerebrospinal fluid (CSF) was sampled, and the animals were sacrificed 42 hours after infection to harvest the brain and serum. Cryosections of the brains were stained for Nissl substance to quantify brain injury. Hippocampal gene expression of Bcl-2, Bax, p53, and BDNF was analyzed. Lithium concentrations were measured in serum and CSF. The effect of chronic lithium treatment on spatial memory function and cell survival in the dentate gyrus was evaluated in a Morris water maze and by quantification of BrdU incorporation after LiCl treatment during 3 weeks following infection. In the hippocampus, LiCl significantly reduced apoptosis and gene expression of Bax and p53 while it increased expression of Bcl-2. IL-10, MCP-1, and TNF were significantly increased in animals treated with LiCl compared to NaCl. Chronic LiCl treatment improved spatial memory in infected animals. The mood stabilizer lithium may thus be a therapeutic alternative to attenuate neurofunctional deficits as a result of pneumococcal meningitis.

## Introduction

Pneumococcal meningitis (PM) causes high mortality and morbidity and leads to persisting sequelae in up to 50% of affected children, including deficits in academic, executive and intellectual performance, which persist into adulthood [1]–[4]. Injury to the brain occurs in 3 specific forms characterized by sensorineural hearing loss due to damage to the inner ear, ischemic necrosis of the cortex and apoptosis in the dentate gyrus (DG) of the hippocampus. The latter from of brain damage is found in patients and experimental models and is regarded as the histomorphologic correlate of learning deficits [5]–[7]. Neuronal apoptosis occurs by caspase-dependent and caspase-independent pathways and is most likely caused by multiple factors including an overshooting inflammatory reaction and bacterial toxins [8], [9].

Lithium is the mainstay treatment of bipolar disorders [10]. Several studies have suggested that lithium can be used in the treatment of acute brain injuries (e.g. ischemia) and chronic neurodegenerative diseases (e.g. Alzheimer’s disease, amyotrophic lateral sclerosis [ALS], Parkinson’s disease [11]–[14]). Lithium is water-soluble, does not bind to plasma proteins and rapidly reaches the brain where it has been described to accumulate in the cells [15]. Clinically, it is administrated in the form of lithium salts, e.g. LiCl and excreted without metabolization by the kidneys where around 80% are reabsorbed in the proximal tube [10]. Lithium serum concentrations have to be closely monitored to prevent toxic effects which are observed above 1.5 mmol/l while neurological symptoms are associated with serum levels >2 mmol/l [16], [17]. Recommendations aim at 0.6–1.0 mmol/l lithium serum concentrations while concentrations above 0.4 mmol/l have been accepted to be effective in chronic treatment of bipolar disorder and are used in studies evaluating lithium in other neurodegenerative paradigms, e.g. ALS [12], [17]–[19]. The exact mechanisms of lithium action remain to be elucidated, but effects on neurotransmitters (e.g. glutamate, dopamine, GABA), second messenger signaling systems (glycogen synthase kinase 3 [GSK-3], inositol), and B-cell lymphoma protein-2 (Bcl-2) have been described [10]. The protein kinase GSK-3β plays a critical role in the central nervous system, is expressed in neurons during remodeling, regulating multiple processes of neurons, including cytoskeletal development to establish polarity [20], [21]. GSK-3β is rapidly and reversibly inhibited by lithium through multiple pathways with an IC_50_∼2 mmol/l, while the mood stabilizing effects are apparent after chronic administration aimed at concentrations of ∼1 mmol/l and may involve different mechanisms [22]–[24]. GSK-3 promotes the mitochondria-mediated intrinsic apoptotic signaling pathway and inhibits the extrinsic apoptotic signaling pathway [22]. Both pathways culminate in caspase activation and eventually apoptosis.

Lithium up-regulates anti-apoptotic molecules such as Bcl-2, brain-derived neurotrophic factor (BDNF) and β-catenin while down-regulating pro-apoptotic molecules like Bcl-2-associated X protein (Bax), tumor protein p53, and caspases [11], [25]. Up-regulation of Bcl-2 was observed in chronically treated rodents with lithium plasma concentrations of ∼0.3 mmol/l [20], [26]. This may be relevant for the treatment of bacterial meningitis, since interventions influencing apoptotic processes have been beneficial in experimental models [8]. Similarly in experimental PM, gene expression of BDNF was enhanced and BDNF administration was neuroprotective [27], [28].

In addition to its neuroprotective properties, lithium is known to enhance neurogenesis and to promote neuronal differentiation of progenitor cells [11], [26], [29]. *In* *vitro*, lithium application and down-regulation of GSK-3β resulted in increased proliferation of adult hippocampal progenitors [30]. The hippocampus is a crucial structure for the formation of episodic and spatial memory and is implicated in emotional behavior and spatial memory [31], [32]. The DG is a stem cell niche with continuous formation of new neurons throughout life and therefore potentially well equipped for repair [33]. PM specifically injures these stem cells and their progenitors and thus permanently impedes endogenous brain repair mechanisms, a fact that may explain the persisting neurofunctional deficits in survivors of PM [34]. Nevertheless, this brain region would, by its neurogenic potential, be well equipped for repair and increased neuronal proliferation is indeed observed shortly after bacterial meningitis, but it is neither sufficient nor sustainable [28], [35], [36]. The Morris water maze evaluates predominantly spatial memory and hippocampal apoptosis is associated with decreased learning performance in this task [37]–[39].

We therefore hypothesized that lithium prevents hippocampal apoptosis in acute experimental PM by down-regulating expression of pro-apoptotic genes, while up-regulating anti-apoptotic genes. Further into the investigation of its effects in PM, LiCl was applied in a clinically relevant setting, i.e. after infection, to evaluate its impact on the neurofunctional outcome. We hypothesized that lithium supports neuronal regeneration by increasing proliferation and survival of neuronal stem/progenitor cells in the damaged hippocampus eventually improving neurofunctional outcome. Thus, we evaluated the survival of new-born cells in the DG during three weeks following PM while the animals were treated with LiCl. Based on its neuroprotective and neuroregenerative properties, this is the first study evaluating lithium as a potential therapeutic strategy in the context of bacterial meningitis.

## Materials and Methods

### Infecting organism

A clinical isolate of *Streptococcus pneumoniae* (serotype 3), was cultured overnight in brain heart infusion medium, diluted in fresh medium, and grown for 5 h to logarithmic phase as reported earlier [40]. Bacteria were pelleted by centrifugation for 10 min at 1′600×*g*, and re-suspended in sterile, pyrogen-free saline to the desired optical density. The accuracy of the inoculum size was routinely confirmed by quantitative cultures on sheep blood agar plates.

### Infant rat model of PM

All animal studies were approved by the Animal Care and Experimentation Committee of the Canton of Bern, Switzerland (license BE 100/11) and followed the Swiss national guidelines for the performance of animal experiments. A well-established infant rat model of PM was used as previously described [40], [41]. Litters of 12 nursing Wistar rats with their dam were obtained from Charles River (Sulzfeld, Germany) and acclimatized for 6 days prior to infection on postnatal day 11 (P11) by intracisternal (i.c.) injection of 10 µl 0.85% NaCl containing log_10_ 5.99±5.78 cfu/ml live *S. pneumoniae* (PM+). Non-infected control animals received i.c. injections of 10 µl sterile 0.85% NaCl (PM−). PM was confirmed by quantitative analysis of bacterial titers in CSF samples, when animals developed symptomatic disease at 18 h post infection (hpi). To assess disease severity, the rats were weighed and examined clinically at 0, 18, 26 hpi, and before sacrificing at 42 hpi. The severity of the disease was scored as follows: 1 = coma; 2 = does not stand upright; 3 = stands upright within 30 seconds; 4 = minimal ambulatory activity, stands upright in less than 5 seconds; and 5 = normal. For ethical reasons, animals reaching a score of 2 or below were sacrificed with an overdose of pentobarbital (Esconarkon, Streuli, Uznach, Switzerland, 150 mg kg^−1^ d^−1^, i.p.); spontaneous mortality was documented. Antibiotic therapy with ceftriaxone (Rocephine, 2×100 mg kg^−1^ d^−1^ i.p., Roche Pharma, Basel, Switzerland) was started at 18 hpi. Animals were provided with water and food ad libitum at natural light cycles.

### Pre-treatment with LiCl in acute PM

Based on previous reports, increasing concentrations of lithium (2, 20, 27, 57, and 63 mg kg^−1^ d^−1^ LiCl, LiDCO, Interdelta AG, Givisiez, Switzerland) were evaluated [15], [42]–[44]. The animals were randomized to receive either subcutaneous (s.c.) injections of LiCl (n = 64) in a volume of 10 µl/g body weight (b.w.), or an equivalent volume of NaCl 0.85% (n = 53) five days prior to infection (P6) as described above. Punctures of the cisterna magna were performed to obtain CSF samples using a 30 G needle at 18 hpi and 42 hpi. CSF bacterial titers were determined on blood agar plates or CSF was centrifuged (16′000×*g* at 4°C for 10 min) and supernatants frozen at −80°C until further use. Animals were sacrificed with an overdose of pentobarbital at 42 hpi. Blood samples were obtained by puncture of the right atrium. After coagulation, serum was isolated and stored at −80°C until further use. For histological analysis the animals were perfused with 4% paraformaldehyde (PFA) in phosphate-buffered saline (PBS) prior to remove their brains and post-fix them in PFA. For gene expression analysis the animals were perfused with ice cold PBS, the brains were dissected, the left hippocampi were isolated and stored in RNAlater solution (Life technologies, Carlsbad, CA, USA) for 1 day at 4°C and thereafter at −80°C until RNA isolation, while the right hemispheres were post-fixed in PFA for 24 h and used for histology.

### Lithium measurements by capillary electrophoresis

Serum lithium concentrations were measured approximately 12 h after the last dose [16], [45]. Lithium concentrations were measured in serum and CSF samples using capillary electrophoresis. The assay employed was similar to that of Pascali et al. [46]. Briefly, 25 µl serum or CSF was diluted 10-fold with water and barium served as internal standard. The running buffer contained imidazole (10 mmol/l), 2-hydroxybutyric acid (15 mmol/l), 18-crown-6-ether (7.5 mmol/l), and sodium hydroxide (15 mmol/l) and was adjusted to pH 4.5 with a few drops of 1 M acetic acid. Samples were analysed on a P/ACE MDQ system (Beckman Coulter, Fullerton, California, USA) using a 75 µm ID fused-silica capillary of 60 cm total length at 20°C and an applied voltage of 16 kV. Samples were pressure injected during 5 s at 0.5 psi. Quantitation was based on a 5-level internal calibration (between 0.24 and 1.89 mmol/l lithium) using corrected peak areas detected at 214 nm and the 32 Karat software (Version 7.0, Beckman Coulter). The limits of quantitation and detection for lithium were 0.10 mmol/l and 0.03 mmol/l, respectively.

### Histomorphometrical analysis

Brain damage was quantified in animals sacrificed at 42 hpi as previously described [41], [47]. The brains were fixed in PFA and cryopreserved in 18% sucrose in PBS at 4°C overnight. In animals used for RNA isolation, only the right hemispheres were used for histomorphometrical analysis. Coronal brain cryosections (45 µm-thick) obtained by systematic uniform random sampling were stained for Nissl substance with cresyl violet. Cortical damage was defined as areas of decreased neuronal density or frank cortical necrosis by simultaneous bright-field microscopy and scanned digitized images using the software ImageJ 1.45l (National Institutes of Health, USA, http://imagej.nih.gov/ij). The volume of cortical brain damage was expressed as a percentage of the total cortical volume determined using the Cavalieri principle by investigating 16 brain sections per animal as described in details previously [48]. Histological features of apoptosis (condensed, fragmented dark nuclei, apoptotic bodies) were counted in 4 different slices spanning the hippocampus of both hemispheres (in brains used for gene expression only the right hemisphere) by a person blinded to the experimental grouping. Apoptotic cells were counted in three visual fields in each of the two blades of the DG and a mean value per animal (totally 48 field, 24 fields for brains used for RNA isolation) was calculated.

### Quantitative analysis of cytokines in CSF

A panel of cyto- and chemokines known to be involved in the pathophysiology of bacterial meningitis was selected to document the inflammatory response in the present infant rat model of PM [49]: tumor necrosis factor (TNF-α), interleukin-6 (IL-6), IL-1β, IL-10, monocyte chemoattractant protein 1 (MCP-1), macrophage inflammatory protein 1 α (MIP-1α), interferon gamma (IFN-γ) [50]. The concentration of these analytes was determined in the CSF using microsphere-based multiplex assays (MILLIPLEX MAP Kit, Rat Cytokine/Chemokine Magnetic Bead Panel, Cat. #RECYTMAG-65K; Millipore Corporation, Billerica, MA). 5 µl of CSF supernatant were diluted to a final volume of 25 µl using the provided assay buffer. A minimum of 50 beads per analyte was measured using a Bio-Plex 200 station (Bio-Rad Laboratories, Hercules, CA). Calibration curves from recombinant standards were calculated with Bio-Plex Manager software version 4.1.1 using a five-parameter logistic curve fitting. If the sample concentration was below detection limit, the value of the detection limit as provided by the manufacturer and multiplied by the dilution factor used for statistical analysis, i.e. TNF-α 9.5 pg/mL, IL-6 153.5 pg/mL, IL-1β 14 pg/mL, IL-10 13.5 pg/mL, MCP-1 45 pg/mL, MIP-1α 4.0 pg/mL, IFN-γ 31 pg/mL.

### RNA isolation

RNA isolation was performed as previously described [51]. In brief, hippocampi were put into 1 ml QIAzol Lysis reagent (Qiagen) and immediately homogenized by a rotor-stator homogenizer (TissueRuptor, Qiagen). RNA was isolated using the RNeasy Lipid Tissue Mini Kit (Qiagen), following the manufacturers protocol. To remove contaminating DNA, 20 µl isolated RNA was treated with DNase using the DNA-free Kit (Ambion, Carlsbad, CA, USA). RNA quality and quantity were determined on the Agilent 2100 Bioanalyzer platform (RNA 6000 Nano, Agilent Technologies, Waldbronn, Germany) and validated on the NanoDrop device (NanoDrop, Wilmington, USA). Only RNA with sufficient quality (RIN>8) and purity (OD_260_/OD_280_ nm absorption ratio >1.95) were further used. Absence of genomic DNA and unspecific binding of primers was confirmed in qPCR by not reaching the threshold after 40 cycles in samples without reverse transcription.

### Quantitative real-time polymerase chain reaction (qPCR)

Synthesis of cDNA was performed with the High-Capacity cDNA Reverse Transcription Kit (Applied Biosystems, Foster City, CA), utilizing 1.5 µg of RNA according to the manufacturer’s instructions. The cDNA samples were diluted 1∶10 with RNase-free water and stored at −20°C. QuantiFast Probe PCR kit (Qiagen, composed of Hot Star Taq Plus DNA polymerase and dNTP mix in PCR buffer) and TaqMan Gene Expression Assays (Applied Biosystems) were used to perform quantitative real-time PCR (qPCR) in technical triplicates in 20 µl reaction volume on the Quant Studio 7 Flex station (Applied Biosystems). Non-template controls were used to confirm absence of contaminating DNA. The following primers (Applied Biosystems) were used: *bdnf* (BDNF, Rn02531967_s1), *bcl2* (Bcl-2, Rn99999125_m1), *tp53* (p53, Rn00755717_m1), and *bax* (Rn02532082_g1). Parameters for baseline and threshold-cycle (Ct) settings were kept constant for each gene. To calculate ΔCt of each investigated gene, the gene *rpl24* encoding the L24 ribosomal protein (Rn00821104_g1) was used as normalizer [50], [51]. ΔΔCt values were calculated by subtracting the ΔCt values obtained for LiCl treated animals from the mean ΔCt value obtained for the controls (PM−/NaCl treated animals). Relative fold increases were calculated using the formula 2^−ΔΔCt^ [52].

### Chronic lithium treatment after acute PM

84 animals were randomized for infection (PM+; n = 56; inoculum log_10_ 5.72±5.49 cfu/ml) or mock-infection (PM−; n = 28). Some animals were excluded because the infection was not successful (n = 6) or the animals died between P13 and P35 (PM+/LiCl: n = 4; PM−/NaCl: n = 2). The remaining infected animals (n = 46) received s.c. injections of 57 mg kg^−1^ d^−1^ LiCl (LiDCO; n = 15), 110 mg kg^−1^ d^−1^ LiCl (Lithium Chloride, Molecular Biology Grade, Calbiochem; La Jolla, CA, USA; n = 7) or an equal volume of NaCl 0.85% (n = 24). 25 animals died due to the infection within 42 hpi (57 mg kg^−1^ d^−1^ LiCl: n = 7, 110 mg kg^−1^ d^−1^ LiCl: n = 6, NaCl: n = 12). In the mock-infection group, 10 animals received 57 mg kg^−1^ d^−1^ LiCl (LiDCO), 4 animals received 110 kg^−1^ d^−1^ LiCl (Calbiochem) and 12 animals received an equal volume of NaCl 0.85%. The first dose was given at 22 hpi, the last one 12 h before sacrificing on P35. All animals were treated with ceftriaxone (2×100 mg kg^−1^ d^−1^ i.p.) for 5 days and received pulses of 5-Bromo-2′- deoxyuridine (BrdU, 50 mg kg^−1^, B5002, Sigma-Aldrich, Buchs, Switzerland) at 18, 42, and 66 hpi.

### Neurofunctional outcome (Morris water maze)

Learning performance was evaluated between P31 and P35 in the Morris water maze task after chronic lithium treatment as previously described in all surviving animals [53], [54]. Gross vestibulomotor dysfunction was first tested by Rota Rod. Animals had to stay on the rotating rod (8 rpm; Ugo Basile srl, Comerio, Italy) for more than 30 seconds, otherwise they were excluded from the water maze test (n = 2). Swimming patterns of the rats were registered with the video tracking system EthoVision XT (Version 8.5, Noldus Information Technology, Wageningen, The Netherlands). The water surface was virtually divided into four quadrants. An adjustable platform measuring 16×13 cm was placed in the center of the first quadrant 0.5 cm below the water surface. Three entry zones in the other quadrants were marked outside the pool. The animals were given 3 days to acclimatize in a light cycle of 12 h light–12 h darkness in the water maze experimental room. The animals performed five training trials (TT) per day, with the invisible platform in a fixed position between P31 and P34. Before TT and on P35 one probe trial (PT) without the platform was performed. Facing the tank wall, the rats were put into the water at one of the entry zones determined by randomization. If an animal found the platform within 90 s, it was allowed to stay on it for 15 s before put back to the cage. If the rat did not find the platform within 90 s, it was guided there by hand and was allowed to stay on it for 15 s. Between trials, the animals rested for 45 min. During TT, total time and distance to reach the platform and velocity were recorded. In PT the mean distance to the center of the virtual platform, the time spent in the quadrant that contained the platform, and the number of crossings through the virtual platform was recorded in the first 30 s of each run. The animals were sacrificed on P35 by an overdose of pentobarbital. Blood samples were obtained by puncture of the right atrium and the serum stored at −80°C until further use. The animals were perfused with ice cold PBS. The brains were dissected and fixed in methanol/acetic acid (95∶5) at 4°C.

### Evaluation of BrdU incorporation (immunohistology)

Hippocampal density of BrdU positive cells was quantified in all survivors after receiving chronic lithium treatment for 3 weeks. To exclude any influence of the training during water maze on BrdU density, only animals successfully finishing this task were included. After fixation in a solution of methanol and acetic acid the brains were embedded in paraffin. 10 µm sections were prepared as previously described [34], [55]. Anti-BrdU sheep polyclonal antibody (1∶1000; Abcam, Cambridge, UK) and the secondary goat anti-sheep Cy3-conjugated antibody (1:1,000; Jackson ImmunoResearch Laboratories, West Grove, PA, USA) were used and counterstained with 4′,6-Diamidin-2′-phenylindol-dihydrochlorid (DAPI). For cell density counting, digitized images, acquired using a fluorescence microscope and a charge-coupled device camera, were used (AxioImager.M1, Zeiss, Germany). Using ImageJ software (NIH), the outlines of the dentate gyrus were determined on the DAPI-stained image and superimposed on the BrdU image. After calibration, the surface areas were determined and cell proliferation determined as the number of BrdU positive cells/mm^2^ [34].

### Statistical analysis

Statistical analyses were performed by using GraphPad Prism software (Prism 6 for Windows, GraphPad Software Inc., San Diego, CA). If not stated otherwise, results are presented as mean values ± standard deviation. The D’Agostino & Pearson omnibus normality test was used to discriminate between parametric and non-parametric values. To compare data between two groups an unpaired Student’s t test was used for parametric data. To compare four groups, two-way analysis of variance (2way ANOVA) was used together with Sidak’s multiple comparisons test for post-hoc analysis. Non-parametric groups were pairwise analyzed with a Mann-Whitney test. Mortality rates were calculated using log rank (Mantel-Cox) test for significance based on all successfully infected animals and numbers of animals sacrificed due to ethical reasons (clinical score≤2) or dying spontaneously. For post-hoc power analysis an online tool was used (http://clincalc.com/Stats/Power.aspx). A chi-square test was used to compare animals with cortical injury to littermates without damage (defined as less than 0.5% affected cortex). A two-tailed *P* value of <0.05 was considered statistically significant.

## Results

### Pre-treatment with lithium in acute PM

#### Clinical and inflammatory parameters

**Figure 1:**
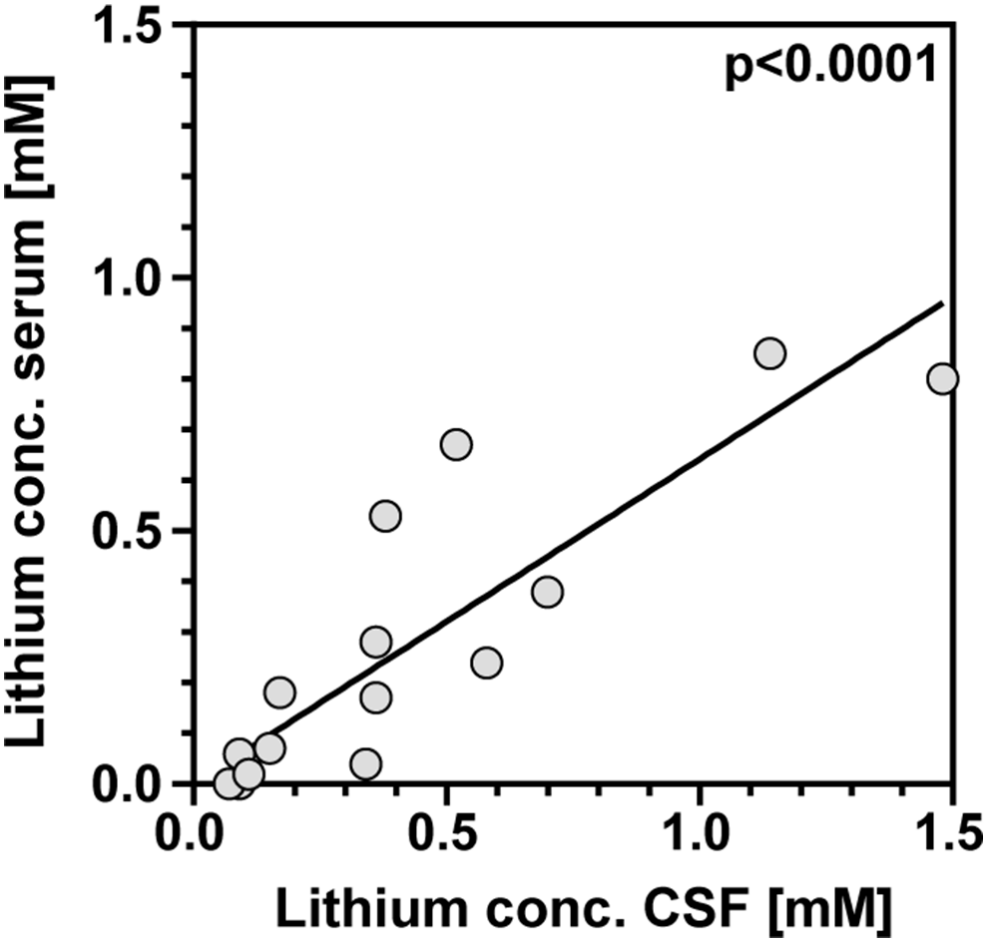
At the time of sacrifice, lithium concentrations in serum and cerebrospinal fluid (CSF) of infected animals show a significant correlation (r = 0.91; p<0.0001; n = 15).

Lithium concentrations were measured in serum and CSF at 42 hpi and clinical and inflammatory parameters were determined to evaluate severity of the disease. Application of 2 mg kg^−1^ LiCl was insufficient to reach measurable serum concentrations of lithium. Therefore, animals with this treatment regimen (n = 36) were excluded from further analyses (except for correlations of hippocampal apoptosis with LiCl dosage and lithium serum concentrations). 53 of 62 rats in the NaCl group and of 64 of 67 rats in the lithium group were successfully infected, which was confirmed by bacterial growth in the CSF at 18 hpi and symptomatic disease, i.e. clinical score <5. Therapeutically effective serum concentrations were defined as 0.4–1.5 mmol/l [17], [18] and were achieved with a dosage between 20 and 63 mg kg^−1^ d^−1^ LiCl. The mean ± SD lithium serum concentration obtained with this treatment regimen was 0.47±0.38 mmol/l (n = 38; range 0.0–1.5 mmol/l) and correlated with the dosage of LiCl applied (r = 0.79; p<0.0001). At the time of sacrifice, in infected animals lithium concentrations in CSF and those assessed in serum correlated (r = 0.91; p<0.0001, n = 15; Fig. 1) and lithium levels in CSF were higher compared to those of serum (slope 0.64±0.06; Fig. 1).

The weight gain during pre-treatment was reduced in animals receiving lithium compared to littermates receiving saline (NaCl: 12.0±1.5 g, n = 53; LiCl: 10.6±1.5 g, n = 64; p<0.0001) and correlated inversely with LiCl dosage (r = −0.40, p = 0.001, n = 64). Consequently, at the time of infection animals receiving lithium weighed less than their littermates (NaCl: 26.2±2.5 g, n = 53; LiCl: 24.9±2.7 g, n = 64; p<0.01). Weight changes between infection and 42 hpi did not vary significantly between treatment groups (NaCl: −0.131±0.052%, n = 34; LiCl: −0.126±0.048%, n = 38; p = 0.50).

At 18 hpi, bacterial titers in CSF were equal between treatment groups (NaCl: log_10_ 7.85±8.08 CFU/ml; LiCl: log_10_ 7.90±7.96 CFU/ml). Survival rates during PM were not significantly changed by lithium pre-treatment (NaCl: 64.1%, n = 53; LiCl: 60.9%, n = 64; p = 0.73).

**Figure 2:**
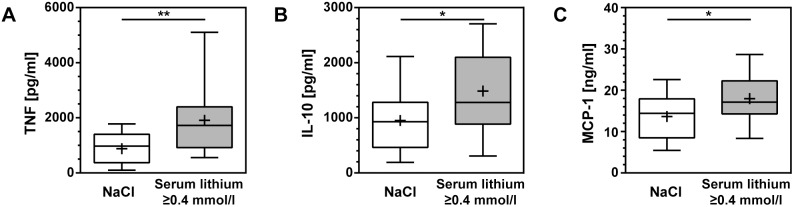
Cyto-/chemokine concentrations in cerebrospinal fluid samples of rats with pneumococcal meningitis and treated with NaCl or LiCl were measured 18 h after infection. All cyto-/chemokines measured were elevated in infected animals receiving LiCl compared to their littermates receiving NaCl. For TNF (**A**), IL-10 (**B**), and MCP-1 (**C**) this difference reached statistical significance. (TNF, tumor necrosis factor; IL, interleukin; MCP-1, monocyte chemoattractant protein 1; boxes extend from the 25th to 75th percentiles and include median; +, mean; whiskers, minimum to maximum value; *, p<0.05; **, p<0. 01).

Cyto-/chemokines were measured in the CSF at 18 hpi (Table 1, Fig. 2). IL-10, MCP-1, and TNF were significantly increased in animals treated with LiCl (n = 17) compared to littermates receiving NaCl (n = 20). Several cyto-/chemokines correlated with serum lithium concentrations measured at 18 hpi: MIP-1α (p = 0.02, r = 0.65), IL-1β (p<0.01, r = 0.75), IL-10 (p<0.005, r = 0.76).

**Table 1:** Cyto-/chemokine levels were measured in cerebrospinal fluid samples of infant rats 18 h after infection.

	NaCl (n = 20)^a^	Lithium (n = 17)^a^^,^ b	P value
**TNF [pg/ml]** c	**877±544 (971, 95, 1780)**	**1905±1166 (1723, 559, 5100)**	0.0012**
**IL-6 [ng/ml]** c	194±100 (211, 34, 354)	193±82.2 (179, 72, 414)	0.8828
**IL-1β [ng/ml]** c	2.0±1.4 (1.7, 0.09, 5.5)	1.5±0.8 (1.3, 0.3, 3.7)	0.2793
**MIP-1α** **[ng/ml]**	6.1±4.0 (6.2, 0.7, 16.5)	6.8±4.4 (6.0, 1.5, 16.6)	0.6020
**MCP-1 [ng/ml]**	**136±51 (144, 55, 226)**	**180±51 (171, 83, 287)**	0.0155*
**IL-10 [pg/ml]**	**952±541 (928, 191, 2114)**	**1486±730 (1279, 306, 2707)**	0.0154*
**IFN-γ [ng/ml]** c	20.2±20.1 (15.8, 0.8, 75.7)	11.5±7.7 (12.7, 0.6, 28.3)	0.2666

a values are mean ± standard deviation (median, min., max.);

b lithium serum conc. 0.4 mmol/l – 1.5 mmol/l;

c non-parametric distribution; *, p<0.05; **, p<0.01; TNF, tumor necrosis factor; IL, interleuki n; MCP-1, monocyte chemoattractant protein 1; MIP-1α, macrophage inflammatory protein 1 α; IFN-γ, interferon gamma.

#### Brain injury

Histomorphometric analysis of hippocampal apoptosis and cortical necrosis was performed on the brains from animals sacrificed at 42 hpi to evaluate neuroprotective effects of lithium. In mock-infected animals (PM−/NaCl and PM−/LiCl) apoptotic cells in the DG were present only sporadically and no injury to the cortex was detected as opposed to infected animals (PM+/NaCl and PM+/LiCl).

**Figure 3:**
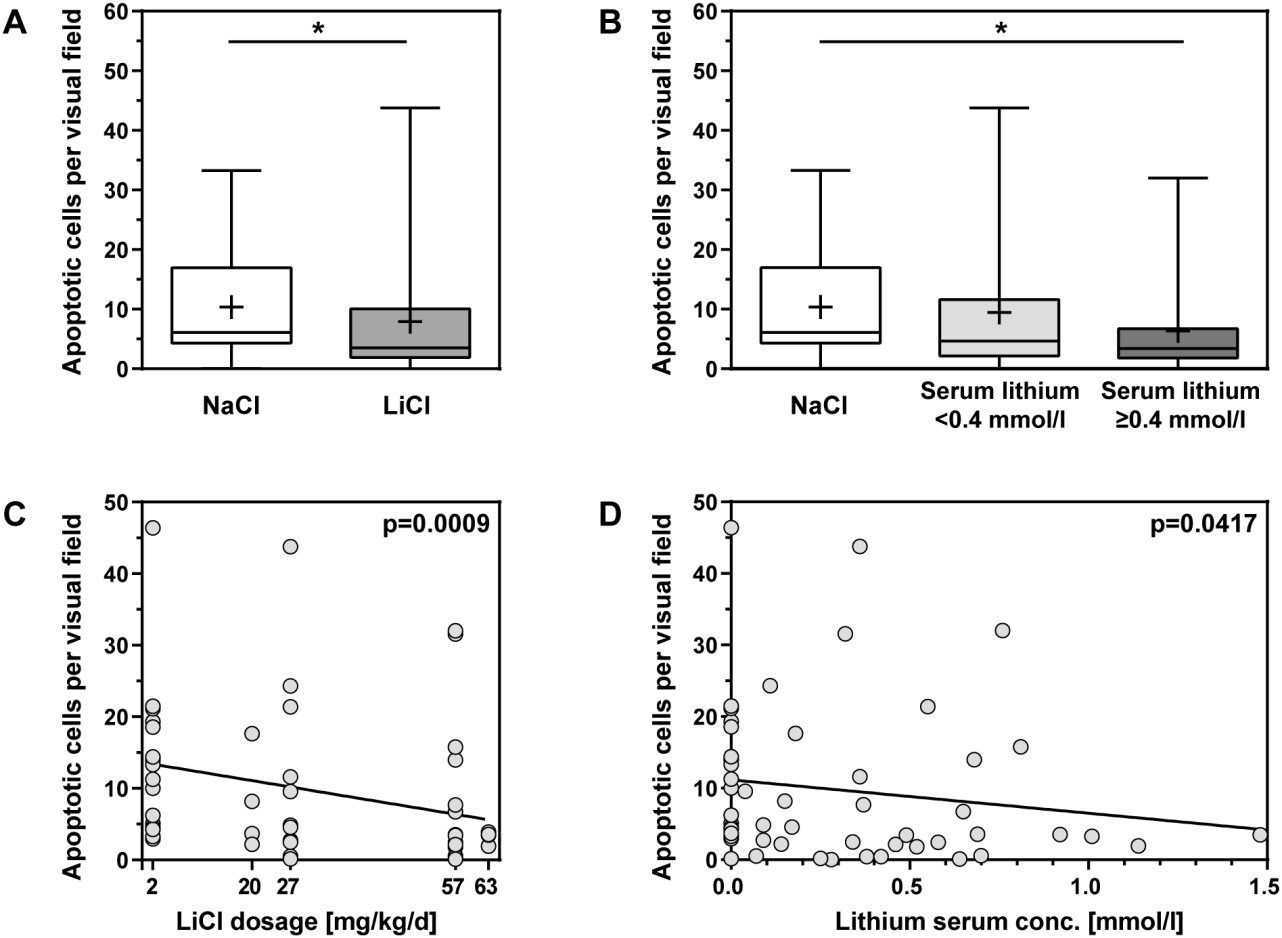
Apoptosis in the hippocampal dentate gyrus of survivors of bacterial meningitis was quantified in cryosections 42 h after infection. (**A**) LiCl treatment reduced apoptosis significantly compared to littermates treated with NaCl. (**B**) Hippocampal apoptosis was significantly attenuated in animals with lithium serum concentrations ≥0.4 mmol/l compared to NaCl treated littermates. (**C**) A dose-dependent effect of LiCl on hippocampal apoptosis is observed (r = −0.43, p<0.001, n = 55). (**C**) Lithium serum concentration and apoptosis in infected rats treated with 2–63 mg/kg LiCl correlate weakly (r = −0.28, p = 0.04, n = 55). (Boxes extend from the 25th to 75th percentiles and include median; +, mean; whiskers, minimum to maximum value; *, p<0.05).

A significant reduction of hippocampal apoptosis was observed in animals treated with a dosage of 20–63 mg kg^−1^ d^−1^ LiCl in comparison to their littermates receiving NaCl (NaCl: 10.4±8.7 apoptotic cells per visual field [c/f], median 6.1 c/f, n = 34; LiCl: 7.9±10.3 c/f, median 3.5 c/f, n = 38; p = 0.03, Fig. 3A). When lithium serum concentrations exceeded 0.4 mmol/l, animals displayed significantly reduced levels of hippocampal apoptosis compared to littermates receiving NaCl (6.4±8.5 c/f, median 3.4 c/f, n = 19; p = 0.03; Fig. 3B). In animals with a lithium serum concentration <0.4 mmol/l this effect did not reach statistical significance (9.5±11.9 c/f, median 4.6 c/f, n = 19; p = 0.48). A dose-dependent effect of lithium could be demonstrated with a significant inverse correlation between the amount of hippocampal apoptosis and increasing LiCl dosages (r = −0.43, p<0.001, n = 55; Fig. 3C). The inverse correlation between measured lithium serum concentrations and the number of apoptotic cells was weaker (r = −0.28, p = 0.04, n = 55; Fig. 3D).

**Figure 4:**
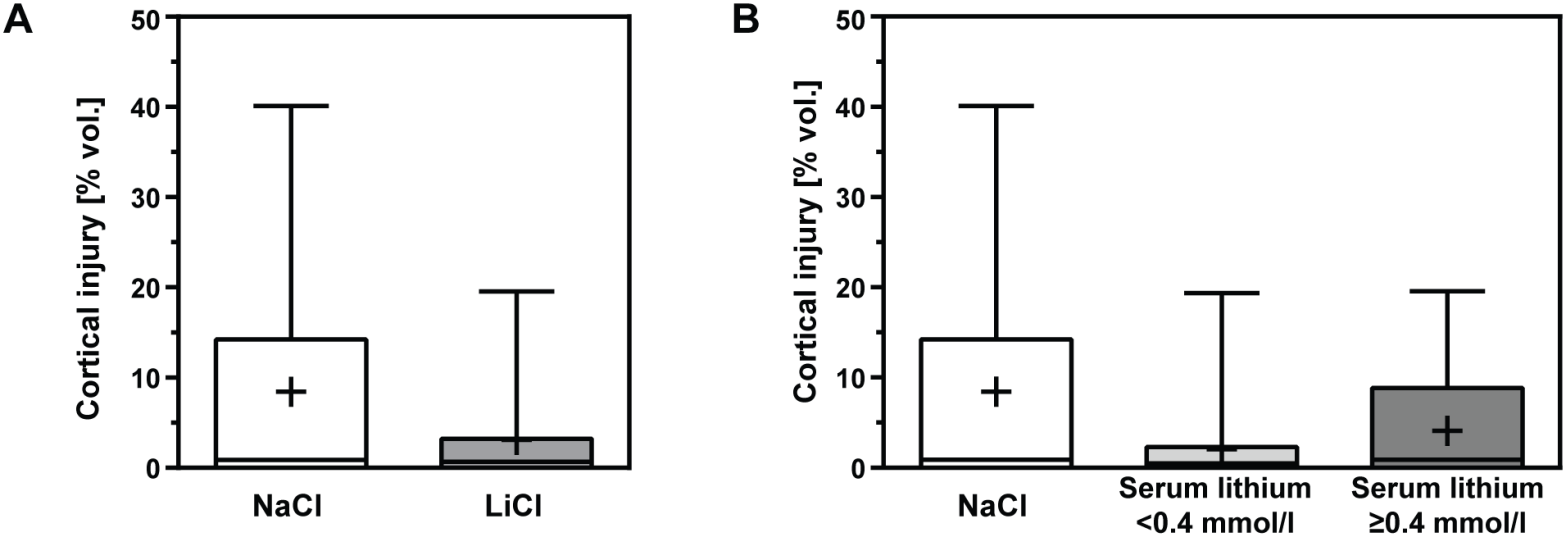
Cortical damage was quantified 42 h after induction of bacterial meningitis in cryosections. (**A**) LiCl treatment reduced cortical injury without reaching statistical significance when compared to littermates treated with NaCl. (**B**) The effect was below statistical significance when comparing animals with lithium serum concentrations ≥0.4 mmol/l to NaCl treated littermates. (Boxes extend from the 25th to 75th percentiles and include median; +, mean; whiskers, minimum to maximum value).

Cortical injury was reduced by LiCl treatment without reaching statistical significance when compared to the NaCl group (NaCl: 8.4±12.2%, median 0.9%; LiCl: 3.1±5.5%, median 0.7%; p = 0.16; statistical post-hoc power: 65.1%; Fig. 4A). Lithium serum concentrations higher than 0.4 mmol/l did not lead to significantly less cortical injury (lithium serum conc. <0.4 mmol/l: 2.1±4.4%, median 0.5%; lithium serum conc. ≥0.4 mmol/l: 4.1±6.3%, median 0.9%; p = 0.80; Fig. 4B). 19 of 40 (47.5%) infected animals treated with LiCl showed cortical injury as compared to 20 of 34 (58.8%) in NaCl treated littermates (Chi square test: p = 0.81).

#### Gene expression analysis

**Figure 5:**
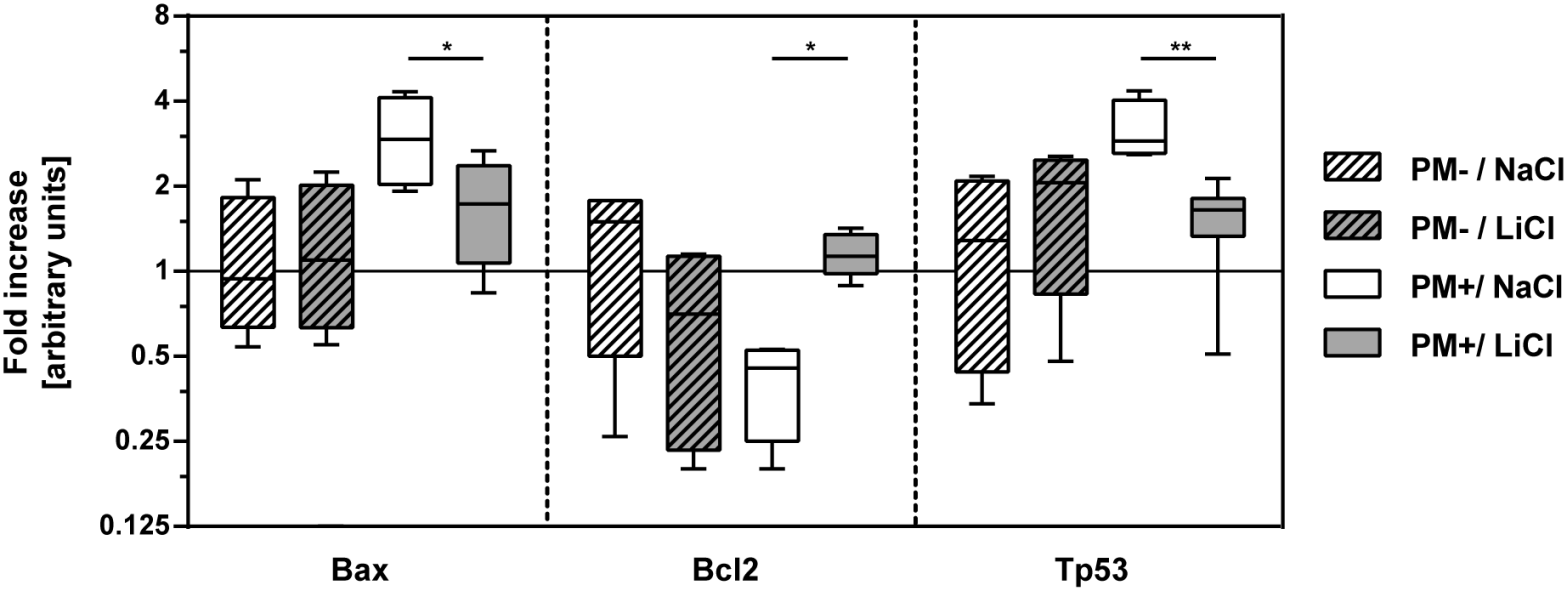
Gene expression of proteins involved in apoptotic pathways was analyzed in hippocampi of 13 days old rats. In mock-infected animals (PM−), gene expression of Bax, Bcl-2, and p53 was not significantly altered by LiCl treatment during 5 days. LiCl treatment significantly reduced gene expression of pro-apoptotic proteins Bax and p53 42 h after induction of meningitis (PM+). Gene expression of the anti-apoptotic Bcl-2 was increased in the LiCl group compared to NaCl. (Boxes extend from the 25th to 75th percentiles and include median; whiskers, minimum to maximum value; *bcl2*, B-cell lymphoma protein-2; *bax*, Bcl-2-associated X protein; *tp53*: tumor protein p53; *, p<0.05; **, p<0.01).

To investigate whether the observed prevention of hippocampal apoptosis could be explained by a direct effect of lithium on apoptotic pathways, expression of *bcl-2*, *bax*, and *tp53* genes was analyzed in the hippocampus of mock-infected and infected animals with and without LiCl treatment at 42 hpi. The *rpl24* gene served as normalizer as described earlier [50], [51]. In mock-infected animals treated with LiCl, expression of the analyzed genes did not change significantly compared to animals. In infected animals expression of *bax* and *p53* genes was significantly up-regulated (p = 0.005, and p<0.05, respectively; 2way ANOVA) compared to mock-infected animals (Fig. 5; Table 2). In infected animals treated with LiCl, *bax* and *p53* gene expression was down-regulated (p = 0.04 and p = 0.01, respectively), whereas expression of *bcl-2* gene was up-regulated (p = 0.02) compared to NaCl treated littermates. In infected animals treated with LiCl, *bdnf* showed decreased expression without reaching statistical significance.

**Table 2:** Expression of genes relevant in apoptotic pathways was analyzed 42 h after infection (PM+) or mock-infection (PM−) by qPCR.

	Treatment(p value^b^)	Infection(p value^b^)	Interaction(p value^b^)	PM−/NaCl(n = 4)^a^	PM−/LiCl(n = 4)^a^	P value^c^	PM+/NaCl(n = 4)^a^	PM+/LiCl(n = 7)^a^	P value^c^
***bdnf***	0.9982	0.7644	0.0052	1.04±0.35	0.26±0.14	0.0724	0.19±0.08^d^	0.97±0.67	0.0647
***bax***	0.1382	0.0054**	0.0801	1.13±0.68	0.25±0.74	0.9738	**3.03±1.09**	**1.75±0.66**	0.0402*
***bcl2***	0.6327	0.3624	0.0040**	1.26±0.72	0.69±0.49	0.1374	**0.41±0.15**	**1.17±0.20**	0.0208*
***tp53***	0.1172	0.0360*	0.0068**	1.27±0.87	1.79±0.91	0.5659	**3.18±0.81**	**1.50±0.51**	0.0051**

a values are mean ± standard deviation (arbitrary units), numbers of animals per group are indicated in parenthesis;

b 2way ANOVA;

c Sidak’s multiple comparisons test;

d n = 3; *, p<0.05; **p<0.01; qPCR, quantitative real-time polymerase chain reaction; *bdnf*, brain-derived neurotrophic factor; *bcl2*, B-cell lymphoma protein-2; *bax*, Bcl-2-associated X protein; *tp53*, tumor protein p53.

### Chronic lithium treatment after acute PM

LiCl was applied in a clinically relevant setting, after the animals developed symptomatic meningitis on the first day after infection. The LiCl dosage applied in the pre-treatment study was used for chronic treatment (57 mg kg^−1^ d^−1^). With this regimen, lithium concentrations measured after 3 weeks of daily LiCl applications at P35 were lower than concentrations measured after 5 days pre-treatment at P13. Therefore, the dose was raised to 110 mg kg^−1^ d^−1^. However, serum levels were not significantly increased under the new treatment schedule at P35. Overall, mean lithium serum concentration was 0.09±0.08 mmol/l (range 0.00–0.21 mmol/l; n = 13) in mock-infected animals and 0.06±0.08 mmol/l (range 0.00–0.22 mmol/l; n = 8) in infected animals measured at P35.

#### Neurofunctional outcome (Morris water maze)

To evaluate the effect of lithium treatment in a clinical setting, neurofunctional outcome was measured in the Morris water maze test 3 weeks after infection in all survivors. Mock-infected and infected animals receiving LiCl during these 3 weeks were compared to their littermates receiving NaCl (PM−/NaCl: n = 12; PM−/LiCl, n = 14; PM+/NaCl, n = 12; PM+/LiCl, n = 9). Gross vestibulomotor dysfunction was excluded by ability to stay on a rotating rod in all survivors, except in two animals of the PM−/NaCl group which were excluded from the study. Learning behaviors, evidenced by decreased distance and time to reach the platform during the training sessions were observed in all four groups over a four-day period.

**Figure 6:**
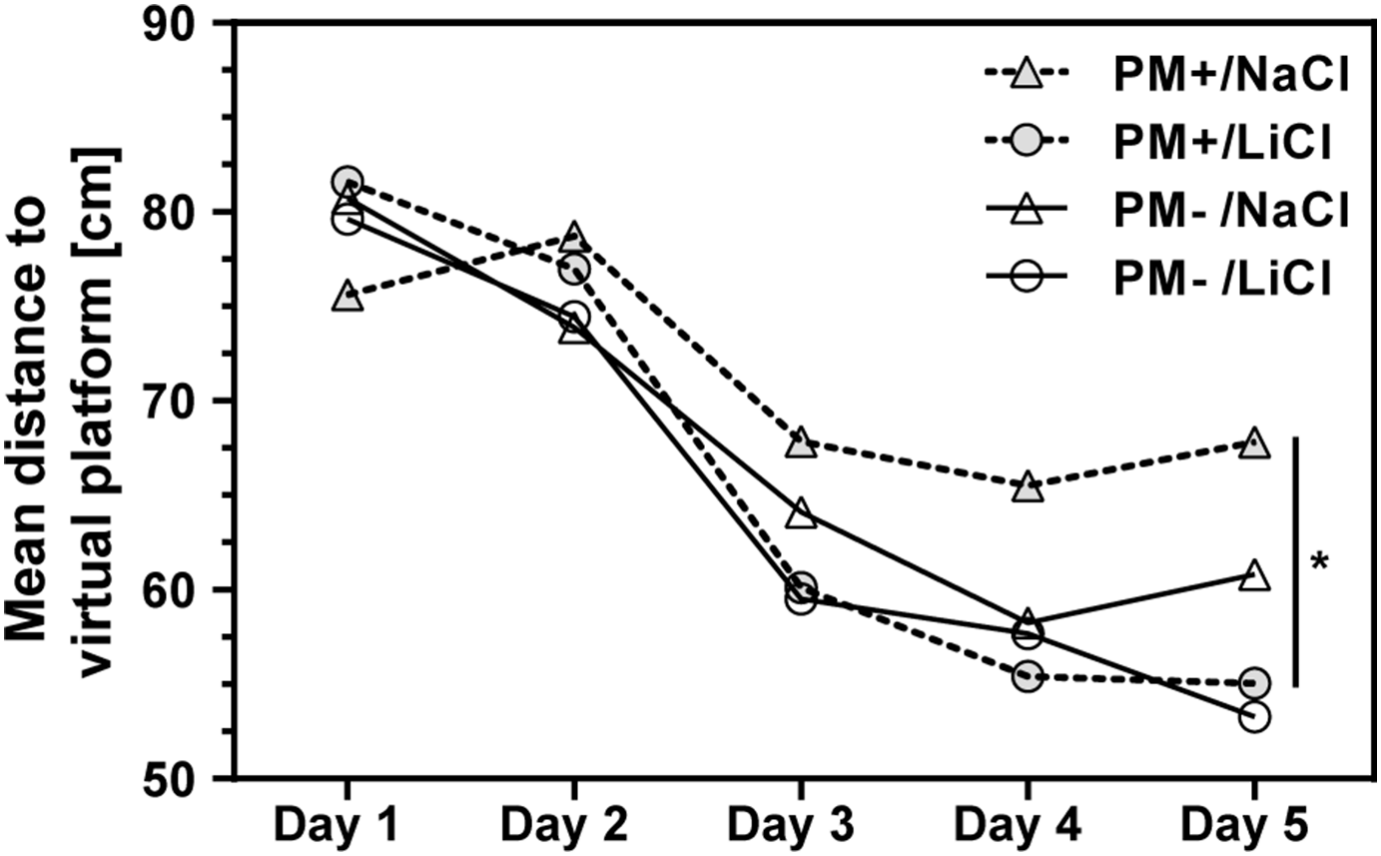
Learning capacity was assessed in the Morris water maze after 4 training days. In probe trials, the mean distance of the animals to the previous location of the platform was calculated. LiCl led to a significantly improved learning capacity compared to NaCl on day 5 (2way ANOVA). In post-hoc analysis, the effect of LiCl treatment was significant in infected animals (PM+) while it remained below statistical significance in mock-infected animals (PM−). (Boxes extend from the 25th to 75th percentiles and include median; +, mean; whiskers, minimum to maximum value; *p<0.05).

Compared to uninfected animals, NaCl treated, infected animals swum a statistically significant longer distance (p<0.005, Mann-Whitney test; Fig. 6) and used statistically significant more time to reach the platform (p<0.01). In contrast, LiCl treated, infected animals did not perform significantly different in this learning assessment than mock-infected ones (distance: p = 0.08; time: p = 0.46). The direct comparison of learning performance between NaCl and LiCl treatment did not reach statistical significance (Table 3).

**Table 3:** Three weeks after infection, the effect of chronic lithium treatment was evaluated in a water maze.

	PM−/NaCl,(n = 12)^a^	PM−/LiCl,(n = 12)^a^	Pvalue^b^	PM+/NaCl,(n = 11)^a^	PM+/LiCl,(n = 8) a	P value^b^
**Time to reach** **platform [s]**	8.3(6.0, 13.5)	9.8(5.7, 15.1)	0.7279	12.4(7.7, 27.4)	11.8(5.9, 23.8)	0.1558
**Distance moved to** **reach platform [cm]**	211.0(146.3, 434.9)	230.8(128.1, 447.4)	0.8276	302.7(196.2, 968.4)	294.6(176.6, 793.3)	0.2530

In training trials, the treatment had no effect on time and distance to reach the platform on day 4.

a values are median (25% percentile, 75% percentile);

b Mann-Whitney test; PM, pneumococcal meningitis.

During probe trials, the platform was removed. Mean distance to the center of the previous location of the platform, number of platform crossings and time spent in the platform’s quadrant were measured. LiCl treatment significantly improved learning performance measured by these three parameters at the end of the learning process, i.e. on day 5 (Table 4). Differences between the treatment groups did not reach statistical significance on days 1 to 4 (data not shown). The effect of lithium treatment was stronger than the effect of infection in the variations between groups reaching statistical significance in the 2way ANOVA on day 5 (Table 4).

**Table 4:** A significant effect of chronic lithium treatment was observed in a water maze after 4 training days.

	Treatment(p value^b^)	Infection(p value^b^)	Interaction(p value^b^)	PM−/NaCl,(n = 12)^a^	PM−/LiCl,(n = 12)^a^	P value^c^	PM+/NaCl,(n = 11)^a^	PM+/LiCl,(n = 8)^a^	P value^c^
**Mean proximity to** **virtual platform [cm]**	**0.0059** **	0.2183	0.4583	60.8±10.8	53.3±12.6	0.1948	**67.8±9.7**	**55.0±12.6**	**0.0422** *
**Platform crossings** **[n]**	**0.0054** **	0.4066	0.8350	1 (0, 5)^d^	2 (1, 5)^d^	0.0805	1 (0, 3)^d^	2.5 (0, 5)^d^	0.0895
**Time in platform** **quadrant [s]**	**0.0262** *	0.3610	0.8717	8.6±3.6	11.3±3.9	0.1221	7.8±2.6	10.1±3.9	0.3071

In a probe trial on day 5 animals treated with lithium swum closer to the previous location of the platform.

a values are mean ± SD;

b 2way ANOVA;

c Sidak’s multiple comparisons test;

d median (minimum, maximum); PM, pneumococcal meningitis;

*p<0.05;

**p<0.01.

#### Survival of new-born cells quantified by BrdU incorporation

**Figure 7:**
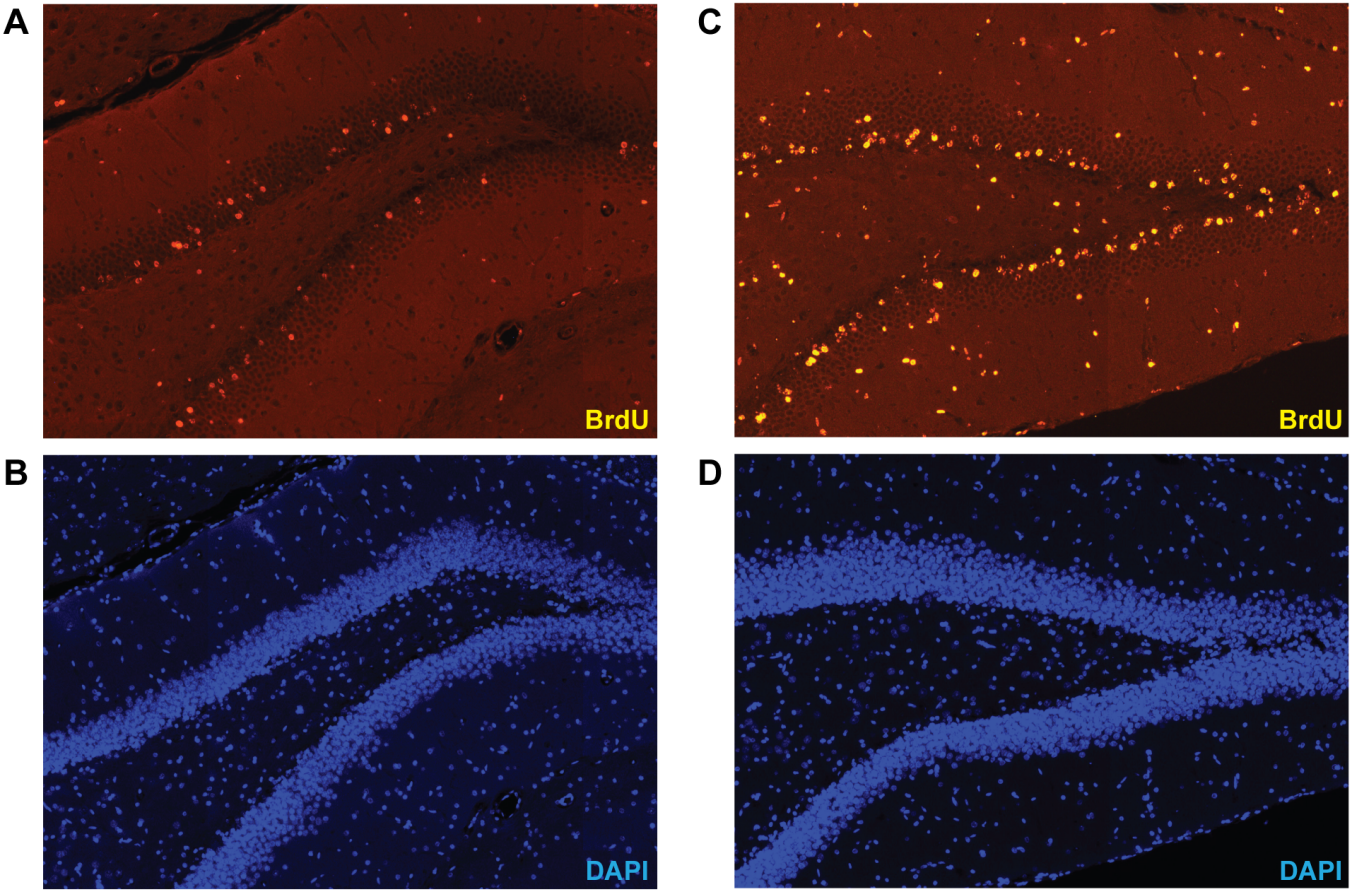
Three weeks BrdU injections to animals with bacteriologically cleared meningitis, BrdU incorporating cells were counted in the dentate gyrus of the hippocampus to quantify cell survival. (**A, B**) Representative image of a mock-infected animal receiving NaCl shows sporadic presence of BrdU positive cells. (**C,** **D**) A mock-infected animal treated with LiCl for 3 weeks shows enhanced presence of BrdU positive cells in the DG. (BrdU, Bromodeoxyuridine; DAPI, 4′,6-diamidino-2-phenylindole).

**Figure 8:**
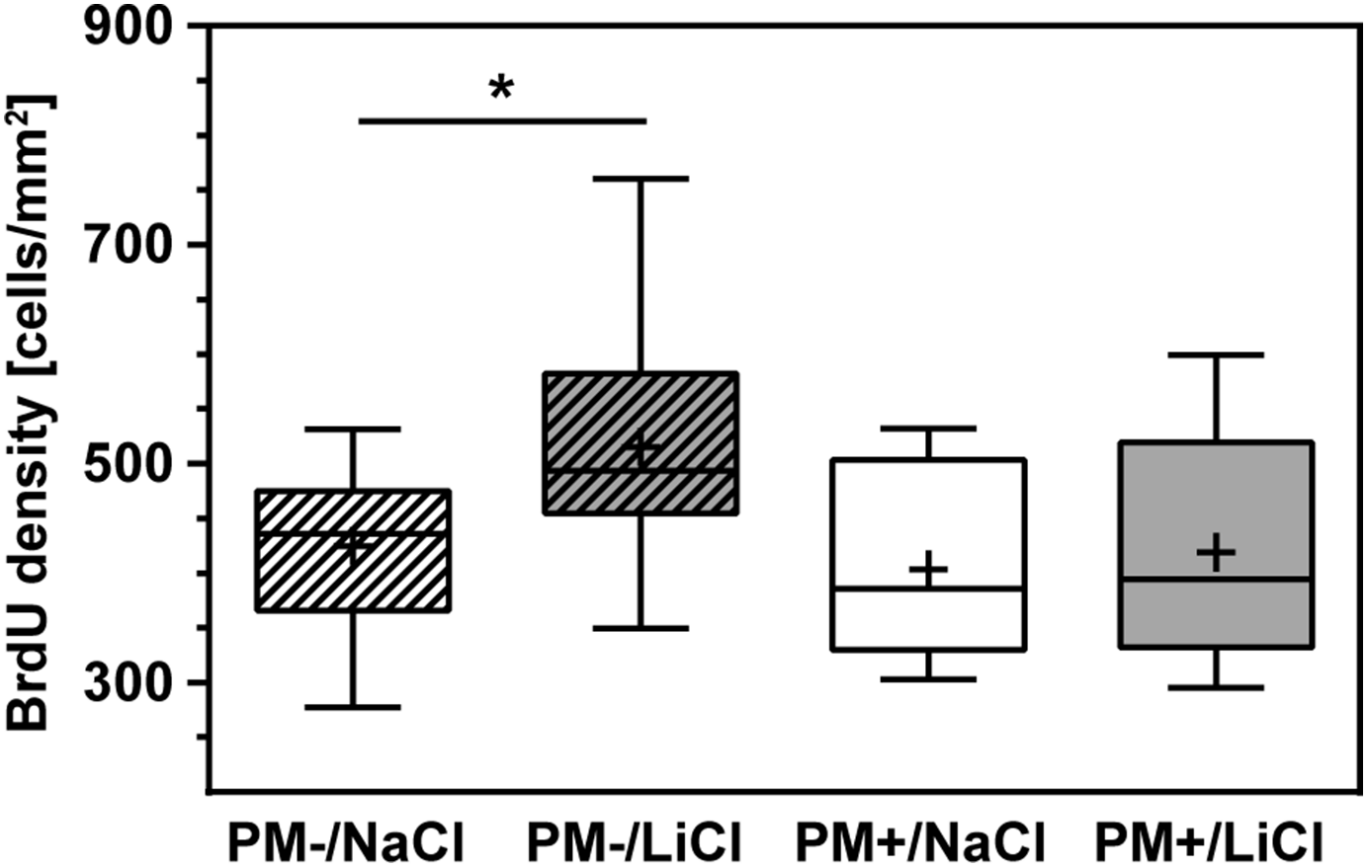
BrdU was applied on P12–14, starting on the first day after infection. The animals were sacrificed on P35 and coronary brain sections were stained for BrdU. In mock-infected animals (PM−), the density of BrdU positive cells in the dentate gyrus was significantly higher after LiCl therapy compared to vehicle, while survival of newborn cells was not altered in infected littermates (PM+). (BrdU, Bromodeoxyuridine; boxes extend from the 25th to 75th percentiles and include median; +, mean; whiskers, minimum to maximum value; *p<0.05).

We hypothesized that chronic lithium treatment after acute PM improves neurofunctional outcome primarily by enhancing proliferation and survival of immature neuronal stem/progenitor cells in the DG [26], [56], [57]. To quantify survival of DG cells that proliferated during and shortly after hippocampal injury due to PM, rats received BrdU pulses 18, 42, and 66 h after infection. The density of BrdU positive cells in the DG was quantified after chronic LiCl treatment of three weeks (Fig. 7). The effect of infection (p = 0.06, 2way ANOVA) and treatment (p = 0.08) remained below statistical significance (interaction: p = 0.22). Post-hoc analysis showed that in mock-infected animals, LiCl treatment significantly increased the survival of BrdU incorporating cells in the DG compared to NaCl application (PM−/NaCl 424.9±73.3, n = 12; PM−/LiCl: 515.1±113.3, n = 14; p<0.05, Fig. 8). In infected animals, LiCl had no influence on the survival of new-born cells in the DG (PM+/NaCl: 395.5±86.3, n = 10; PM+/LiCl 419.3±106.4, n = 9; p = 0.93).

## Discussion

To evaluate the effects of lithium treatment it was necessary to find a valid treatment regimen. In the infant rat model of PM, 57 mmol/l LiCl was most effective to reach desired lithium serum and CSF concentrations during pre-treatment, eventually preventing hippocampal brain damage. However, a slightly reduced weight gain in LiCl treated animals was observed during pre-treatment, which could be explained by polyuria, a known adverse effect of lithium [10], [58]. The higher lithium levels found in CSF compared to serum 42 h after infection might be explained by an opened blood-brain barrier during neuroinflammation compared to earlier studies performed in healthy animals [15], [59].

Survival rates during acute PM were similar in animals receiving lithium and littermates treated with NaCl. Clinical disease severity and mortality are mainly influenced by systemic manifestations of infection, e.g. sepsis, while the direct influence of brain injury on these parameters is weak [60]. The effect of lithium on inflammation is debated and its influence on mortality during acute PM was expected to be weak [61]. In the infant rat model of PM, most animals die within a time window of 20 and 27 hpi, which was also the case in the present study [41]. Therefore, the effect of chronic lithium treatment (initiated at 22 hpi) on mortality rates was expected to be small. Mortality rates can vary considerably in the animal model (inter-litter variability), but they remained within expectation in the present study. After bacteriologically cleared PM, the mortality rate was slightly higher in animals chronically treated with LiCl compared to NaCl treated littermates. We cannot rule out that temporary high lithium CNS concentrations contributed to the death of these animals.

### Lithium influences apoptotic pathways and prevents hippocampal brain damage

Apoptotic injury in the DG of the hippocampus is a neuropathologic hallmark of PM and starts to appear around 18 to 24 h after intracisternal infection in experimental models. It is the result of a multifactorial process which includes an excessive immune reaction to invading pneumococci and bacterial products released in the CSF, while usually no direct contact of the bacteria or infiltrating leukocytes with the DG is observed [7], [8]. Different approaches to reduce brain injury in PM by modulation of the apoptotic machinery have been described earlier, but this is the first study investigating the mood stabilizer lithium in a paradigm of acute neuroinfection [8]. Neuroprotective effects of lithium were described in *in* *vitro* experiments, where the up-regulation of anti-apoptotic Bcl-2 and down-regulation of pro-apoptotic p53 and Bax were observed [11], [62]. In mice, an increase of Bcl-2 in the hippocampus was determined after 3–4 weeks of treatment with Li, together with an increase in the number of dividing cells in the DG [26]. Neuronal apoptosis due to hippocampal irradiation was reduced in adult mice pre-treated for 7 days with lithium [63]. We therefore hypothesized that lithium prevents apoptotic brain injury in experimental PM by modulating expression of genes involved in apoptotic pathways.

In the present study, pre-treatment for 5 days with LiCl successfully reduced apoptosis in the hippocampal DG in an infant rat model of PM. In this paradigm, LiCl had an anti-apoptotic effect by decreasing gene expression of *bax* and *tp53* while increasing expression of *bcl-2* in the hippocampus.

In the subset of animals randomly chosen for gene expression analysis, the protective effect on LiCl on hippocampal apoptosis was less pronounced than that of the overall study-population. The effect on gene expression may therefore be more pronounced in the total population of animals treated with lithium, in which the prevention of apoptosis was more important.

The effect of LiCl treatment on gene expression in mock-infected animals was only marginal. In the present study, CSF levels of lithium were measured in infected animals, characterized by an increased permeability of the blood brain barrier. It is therefore conceivable that in non-infected animals the lack of an effect of lithium on gene expression may be due to lower central nervous system lithium levels compared to those of infected animals. Alternatively, the dysregulated gene expression caused by neuroinfection might have been down-modulated by the effect of lithium.

Independently of LiCl treatment, the infection did not significantly alter gene expression of BDNF at 42 hpi. During bacterial meningitis, elevated levels of BDNF in serum and CSF have been reported [28], [64]. Different *in* *vitro* and *in* *vivo* models showed increased BDNF expression after chronic treatment with lithium, i.e. 14 days and longer [11], [65], [66]. Furthermore, increased RNA and protein levels of BDNF were described in a mouse model of PM only 4 days, but not 30 h after infection [28]. This is in agreement with a microarray analysis performed in infant rats with PM, showing that neuroregenerative processes are initiated 3 days after infection [55]. Therefore, we cannot exclude that the infection regulates BDNF expression at later time points than the one we tested in the present study.

### Lithium increases cyto-/chemokines in CSF while it has little effect on cortical injury

In PM, the mechanisms leading to cortical damage are multifactorial and mostly related to inflammation and/or oxidative damage and bacterial products [8], [41]. In a model of cerebral ischemia, reduction of infarct size and improved neurological outcome after pre-treatment for 16 days with lithium has been described [67]. In the present study, injury to the cortex was only modestly reduced in lithium treated animals. Whether reduced inflammation observed in some other experimental settings is a primary effect of lithium application or results from less injury is unknown [63]. Lithium has been described to act both, pro- and anti-inflammatory and it is known to induce leukocytosis [61]. In the present study, significant up-regulation of TNF, IL-10, and MCP-1 during acute PM was observed in lithium-treated animals, indicating a higher degree of inflammation while IL-1β and IFN-γ were down-regulated without reaching statistical significance. Other reports described increasing IL-10 and decreasing TNF concentrations after lithium treatment [61]. However, the effect of lithium on these and other cytokines varied under different conditions [61]. For example, one study reported increased TNF secretion by neutrophils after lithium treatment during an acute inflammatory reaction. In this study, mRNA levels of TNF were not altered, indicating a post-transcriptional regulation [68].

TNF is an early response cytokine triggering an intense immune response and has been targeted in meningitis models as a therapeutic approach [41]. Though, inhibition of TNF activity may be a double-edged sword and interventions aimed at specific immunological mechanisms need to be well balanced [8]. Lithium may increase inflammation locally, evidenced in the present study by raised cyto-/chemokines in CSF samples, while having specific anti-apoptotic properties and preventing hippocampal damage, but not cortical necrosis.

In summary, LiCl was able to reduce apoptosis in the hippocampus by favorably modulating the expression of genes involved in the apoptotic machinery. In contrast, LiCl treatment had no impact on weight loss or clinical score. Furthermore, inflammation was not attenuated by LiCl administration and no significant effect on cortical damage could be observed. These proof-of-concept experiments were the basis to investigate the effects of lithium in an adjuvant setting with a clinically relevant treatment regimen.

### Chronic lithium treatment after PM improves spatial memory

The dosage used for pre-treatment (57 mg kg^−1^ d^−1^) was applied in the study evaluating chronic lithium treatment starting at 22 hpi, and resulted in serum levels of max. 0.22 mmol/l at P35. An increase to 110 mg kg^−1^ d^−1^ did not increase serum concentrations at P35, although lithium serum concentrations of 1.97 mmol/l and 2.64 mmol/l were measured in two infected animals euthanized on P16. An effect of LiCl therapy, at the measured serum concentrations, may be due to the higher lithium serum levels that are reached at a younger age since serum concentrations primarily depend on renal function which is lower in infant rats. This is due to the fact that lithium is not metabolized or bound to any proteins and urinary concentrating ability is fully developed at around 6 weeks of age [45], [69], [70]. Alterations of renal clearance, volume of distribution or other adaptations in the infant rat may be responsible for the different lithium serum concentrations measured at a different age.

In the present study in rats that survived PM, lithium led to significantly improved learning performance during probe trials compared to NaCl. The time and distance to reach the platform during training trials decreased in all groups, without reaching statistical significance when comparing animals with LiCl therapy to their littermates receiving NaCl. In earlier studies, chronic lithium treatment improved spatial memory assessed in a water maze during different conditions, e.g. traumatic brain injury [71]. A correlation between hippocampal neurogenesis and learning has been described earlier [72], [73]. Lithium has been shown to increase neurogenesis, e.g. evidenced by enhanced BrdU labelling of cells in the DG and double-labelling with NeuN [19], [26], [56]. Also, an effect on long-term potentiation has been observed earlier [57]. Immature neurons appear to become involved in spatial memory at 15–20 days of age in rats [33]. Most immature cells die within the first 2 weeks after proliferation, while training during days 6–10 following BrdU injection enhanced survival [33]. PM increases the proliferation of neuronal progenitor cells in the first week after infection with a peak around 2 days post-infection and thereafter declines to basal levels [36], [55]. However, this increase in proliferation does not prevent learning deficits. In the present study, this issue was addressed by daily applications of lithium over 3 weeks, starting on the first day after infection.

The effect of chronic lithium treatment on the survival of cells born in the dentate gyrus 1 to 3 days after infection was evaluated 3 weeks later. While LiCl increased cell survival in mock-infected animals, lithium therapy was not sufficient to prolong survival of new-born cells in infected animals. Thus, the observations made during neurofunctional assessment cannot be explained solely by lithium-induced survival of new cells in the DG (neurogenesis). Lithium’s ability to mobilize stem cells may contribute to the observed beneficial outcome [74], [75]. Furthermore, GSK-3 has a critical function in regulating axon genesis and elongation and inhibition by lithium stimulated axon formation and elongation of mature neurons *in* *vitro* and in an *in* *vivo* model of spinal cord injury [21]. Enhanced functional integration of surviving neurons might be an alternative to explain the observed difference in the water maze. Indeed, a recent study showed improved functional recovery by lithium after intracerebral hemorrhage in rats without affecting BrdU incorporation and doublecortin staining in the DG [76]. We also cannot rule out that only the first doses of LiCl applied during the very first days after infection may have acted neuroprotective rather than neuroregenerative, explaining the preserved learning capacity 3 weeks later. However, since steady state levels of lithium are only reached after 1 week, i.e. later than the development of brain damage, a neuroprotective effect of lithium by chronic administration beginning at the onset of the symptomatic disease is unlikely [10].

One of the main difficulties for lithium treatment is the narrow therapeutic range of serum and CSF concentrations, while steady concentrations are reached only after one week [10]. Furthermore, the mechanistic functions of lithium at different concentrations are widely unknown and lithium concentrations varied in animals with a different age. We have addressed this issue by measuring CSF concentrations at the time of sacrificing, however a continuous observation of serum and CSF concentrations would be desirable. Within the current study design it was not possible to explain the beneficial effect of lithium on spatial memory.

## Conclusion

Herein, lithium has shown a neuroprotective effect on hippocampal brain damage when administered in the acute disease. When given for a prolonged period after the disease, lithium led to improved spatial memory. Thus the mood stabilizer Lithium may be a potential strategy to prevent neurologic sequelae in consequence of PM.
